# Identification of Differentially Expressed Genes Relevant to Corm Formation in *Sagittaria trifolia*

**DOI:** 10.1371/journal.pone.0054573

**Published:** 2013-01-24

**Authors:** Libao Cheng, Shuanyan Li, Xiaoyong Xu, Javeed Hussain, Jingjing Yin, Yi Zhang, Liangjun Li, Xuehao Chen

**Affiliations:** 1 School of Horticulture and Plant Protection of Yangzhou University, Yangzhou, Jiangsu, People’s Republic of China; 2 China-UK HUST-RRes Genetic Engineering and Genomics Joint Laboratory, College of Life Science and Technology of Huazhong University of Science and Technology, Wuhan, Hubei, People’s Republic of China; Wuhan University, China

## Abstract

*Sagittaria trifolia* is a good model of wetland plants to elucidate the formation of corm. However, few studies have been conducted to uncover the complexity of gene expression involved in corm formation. In this study, high-throughput tag-sequencing based on Solexa Genome Analyzer Platform was applied to monitor the changes in gene expression with three libraries of differentially expressed genes (DEGs) (C1 library: stolon stage, C2 library: initial swelling stage and C3 library: swelling stage) during corm formation in *Sagittaria trifolia*. Approximately 6.0 million tags were sequenced, and 5854021, 5983454, and 5761079 clean tags including 138319, 116804, and 101739 distinct tags were obtained after removal of low quality tags from each library, respectively. About 46% distinct tags were unambiguous tags mapping to the reference genes, and 33% were unambiguous tag-mapped genes. Totally, 20575, 19807, and 18438 were annotated in C1, C2, and C3 libraries, respectively, after mapping their functions in existing databases. In addition, we found that profiling of gene expression in C1/C2 and C2/C3 libraries were different among most of the selected 20 DEGs. Most DEGs in C1/C2 libraries were relevant to hormone synthesis and response; energy metabolism and stress response, while most of the genes in C2/C3 libraries were involved in carbohydrate metabolism. All up-regulated transcriptional factors and 16 important genes relevant to corm formation in three libraries were also identified. To further analyze the expression of 9 genes, from the results of tag-sequencing, qRT-PCR was applied. In summary, this study provides a comprehensive understanding of gene expression, during the formation of corm in *Sagittaria trifolia*.

## Introduction

*Sagittaria trifolia*, a member of the family Alismataceae, is an aquatic herb vegetable and widely cultivated in China. The corm of *Sagittaria trifolia* is very popular in the daily diet because of its richness in nutrients including starch, proteins, vitamins, and mineral substances [1]. China exports the processed products of *Sagittaria* to Asian countries as a kind of off-season vegetable [2]. In addition, it is also an important ingredient in the traditional medicine in treating wounds, headaches, indigestion and rheumatism [3].

During the whole growth season from March to October, *Sagittaria trifolia*, like most wetland species, is grown in the shallow water, such as the pools, water gardens, tanks or tubs in the greenhouse, which indicates that the plant has developed mechanisms of surviving in the submerged environment. For the regeneration of this plant, bare root stocks or seedlings are directly planted into wetland soil. Several buds from the main stem develop into stolons, and the corms are formed at the tips of each stolon [4]. Actually, corm, tuber, rhizome and bulbs are kinds of underground stems, and work as storage organs. These are storage units for food that provide the plants with the energy for growth, blooming, and completing their lifecycle.

The process of corm formation (similar with potato) can be classified into three stages: induction, initial swelling, and swelling stage [5]. Stolon tips grow radially in the induction stage. In the second stages, longitudinal growth of stolon stops and its tips swell [6]. At this swelling stage, great amounts of carbohydrates are synthesized in the storage. Starch content in *Sagittaria trifolia* corm consists of 10–20% of total fresh weight. Essentially, all above processes are controlled by the signal molecules and environmental factors [7], [8].

Swelling processes of underground plant storage organs (tuber, corm, rhizome, and bulb) have been extensively studied in tuber as compared with other underground stems (corm, rhizome, and bulb). Great changes have been found in genetic and morphometric processes during the formation of storage organs [9], [10].

Similar with tuber development of potato, corm formation of *Sagittaria trifolia* is also under a strict photoperiodic control. The formation of corm is promoted in short days (SD), but prolongs in long days (LD) [11]. It is reported that the signal of photoperiodic control is perceived by the leaf, and then transported via the phloem to the vegetative shoot apex or underground stolon tips, which promotes the transition of storage organ. *Flowering Locus T*, *LKP2*, *CONSTANS* and *GIGANTEA* has been found to be involved in the signal transduction of photoperiodic control, and these gene expressions affect the formation of storage organ [12], [13], [14], [15].

Chen et al. (2004) find that formation of storage organ is promoted by StBEL5 and KNOX through repressing the gibberellin StGA20ox1 biosynthesis under SD condition [16]. At the same time, the expression of StBEL5 is enhanced by miR172, suggesting that long distance transport of RNA signal also participates in the formation of underground storage organ [17]. In addition, PHYB is also involved in the formation of storage organ in SD. Decreasing the levels of PHYB in transgenic plants lead to the formation of storage organ both in SD and LD. Compared with transgenic plants, control can form storage organ in SD [18], suggesting that plants lose the inhibitory effect on tuberization caused by LD [12]. At present, high sucrose content is reported to be the optimal condition required for the formation of storage organs [12]. During the early stages of storage organ development, it requires an active sucrose transporter to trigger the formation of storage organ [19], which indicates that the role of sucrose is necessary for the formation of storage organ at the initial swelling stages [20].

Evidence shows that several phytohormones including: gibberellic acid (GA), cytokinin, jasmonic acid, abscisic acid (ABA), indole acietic acid (IAA), ethylene and jasmonate are also involved in the initiation and regulation of growth in these storage organs [6], [21], [22], [23]. It has been reported that exogenous application of GA acts as an inhibitor of tuber induction. Overexpression of GA oxidase gene in transgenic potato plants postpones the tuber development. Whereas, inhibition of this gene results in an early tuberization than wild type plants [19], [24]. Cytokinin and jasmonic acid promote the tuber induction and elongation [25]. Bhat et al [26] found that exogenous cytokinin is necessary to induce formation of tuber in ginger due to improvement of photosynthesis. ABA shows high correlation to tuber formation because ABA-deficient potato plants show retarded tuberization [27]. Exogenous application of auxin on the decapitated peas and potatoes inhibits the formation of axillary buds [28]. Ethylene, produced by almost all plants mediates a variety of developmental processes in plants, such as seed germination, lateral bud stimulation, adventitious rooting, overcoming dormancy and organ senescence and abscission [23], [29]. Exogenous ethylene is believed as an inducer for the tuberization in potato and root bulking in carrots [30].

Just like the other storage organs, corm of *Sagittaria trifolia* is also an important edible product, and the developmental processes of this kind of storage organ is regulated by many genes [8]. These differentially expressed genes (DEGs) promote the formation of corm. Although much work in other species has partially described the above processes, expression of genes which affect corm formation in *Sagittaria trifolia* is has not been studied in detail. Especially in potato, many tuber related genes have been documented [31]. High throughout transcriptome assembly has been established as an efficient approach to study gene expression in different environmental conditions [32], [33]. A lot of important genes involved in plant critical metabolisms have been successfully identified from horticultural species, such as cucumber, potato, tomato, and Chinese cabbage [34], [35], [36], [37]. In this study, DEGs from three developmental stages of *Sagittaria trifolia* corm were sequenced and analyzed with aim to comprehensively understand the processes of corm formation at molecular level. qRT-PCR method was also applied to evaluate expression characteristics of some genes involved in corm formation.

## Materials and Methods

### Plant Materials

*Sagittaria trifolia* cv. Sinensis which is commonly cultivated in China for daily diet, was planted in the field with water depth of 20 cm in spring with average temperature 25°C/day and 17°C/night during the whole growth season. Several stolons developed and elongated in proper order in each plant. When plants grew up to 8–10 leaves stage (about 100 days after plantation), formation of corm started at stolon tips. Corms of three developmental stages (stolon, initial swelling, and swelling stage) from the plants (five tips from different plants were combined for each stage) were used for analysis of tag-sequencing and gene expression. To get the materials of different developmental stages, plants were cultivated in a field (non-private), located in the South-Eastern China. The permission for sample collection was taken from the Department of Horticulture of YangZhou University, China. No specific permissions were required for the location and the field studies, because the experiments did not involve any endangered or protected species.

### Screening the DEGs

Corm transcriptome from the above three development stages was analyzed. Stolon tips, corms in the initial swelling and swelling stages were collected and ground, and the RNA was isolated from the ground samples using RNA extraction mini kit (QIAGEN, Germany). DNaseI was added to eliminate DNA contamination. Sequencing of the transcripts in the form of special constructs was completed by Beijing Institute of Genomics (BIG).

To screen the DEGs, transcriptome from these three stages was analyzed with the aspirations to track the major changes in the metabolism. RNA was isolated from the materials of these three stages. The DEG libraries of the three samples were determined in parallel using Illumina gene expression sample preparation kits. Briefly, the total RNA from three stages was used for mRNA capture with magnetic oligo (dT) beads. The first and second strand cDNA were synthesized, and bead-bound cDNA was subsequently digested with *NlaIII*.

The 3′-cDNA fragments attached to the oligo (dT) beads were ligated to the Illumina GEX NlaIII adapter 1, which contained a recognition site for the endonuclease MmeI for cutting 17 bp downstream of the recognition site (CATG) to produce tags with adapter 1. After removing the 3′ fragment via magnetic beads precipitation, an Illumina GEX adapter 2 was introduced at the site of MmeI cleavage. The resulting adapter-ligated cDNA tags were amplified using PCR-primers that were annealed to the adaptor ends for 15 cycles.

The 85 base fragments were purified and recovered by 6% polyacrylamide Trisborate-EDTA gel. The final quality of the tagged sequences was checked by an Agilent 2100 Bioanalyzer. The three tag libraries constructed underwent Illumina proprietary sequencing chip for cluster generation through *in situ* amplification and were deep-sequenced using Illumina Genome Analyzer. For the raw data, we filtered adaptor sequences, low quality tags (tags with unknown nucleotides N), empty reads and tags that were too short or too long, and tags with only one copy to get clean tags. The types of clean tags were represented as the distinct clean tags. Subsequently, we classified the clean tags and distinct clean tags according to their copy number in the library, and showed their percentage in the total clean and distinct tags, and analyzed saturation of the three libraries.

For annotation, all tags were mapped to the reference sequence of NCBI database (http://www.ncbi.nlm.nih.gov/), and no more than 1-bp nucleotide mismatch was allowed. The alignment procedures were conducted essentially by following the protocols described in the online documentation (http://maq. sourceforge.net) and adopting the default parameter values. To monitor mapping events on both strands, both sense and complementary antisense sequences were included in the mapping process. The tags mapped to reference sequences from multiple genes were filtered [34].

### Identification of DEGs

The transcriptome of the *Sagittaria trifolia* from the above three stages was used as reference for the screening and analysis of the DEGs due to unavailability of the existing data. All expressed genes were monitored, and the gene functions were explored by using database annotations like nr, Swiss-Prot, KEGG, and COG with following criteria: for the gene annotations, blastx alignment (evalue<0.00001) between unigenes and protein databases, such as Swiss-Prot, KEGG, and COG were performed. Best aligning results were used to decide sequence direction and functions of the unigenes.

In case of any conflict in the results from different databases, a priority order of nr, Swiss-Prot, KEGG, and COG was followed when deciding sequence direction of unigenes. When a unigene happened to be unaligned with none of the above databases, ESTscan was used to predict its coding regions as well as to decide its sequence direction. All of the expressed unigenes were classified according to their functions in metabolism processes. For screening the differentially expressed genes, “FDR≤0.001” and the absolute value of “log_2_ Ratio ≥1” were used as a threshold to judge the significance of difference in expression of unigenes.

### Gene Expression Analysis by qRT-PCR

Quantitative RT-PCR analysis was performed to quantify the transcriptional level of nine novel genes associated with corm formation. Total RNAs were extracted using RNA extraction mini kit (QIAGEN, Germany) from stolon tips, corms of initial swelling stage, and swelling stage respectively. DNaseI was used to digest DNA during the RNA extraction process to eliminate DNA contamination. A total of 1–2 µg of RNA was used in cDNA synthesis according to the manufacturer’s instructions (Promega, USA). The quantitative RT-PCR reaction was performed with the Mx 3000P machine (STRATAGENE, http://www.stratagene.com). The SYBR Green Master Mix was used to identify mRNA level according to the manufacturer’s instructions (Tiangen, China).

According to the results of sequencing, the primers used for nine genes relevant to corm formation were designed which are listed in Table 1. *β-Actin* of *Sagittaria trifolia* was used as internal standard and amplified with the primers, forward: 5′-AACCTCCTCCTCATCGTACT-3′, and reverse: 5′-GACAGCATCAG CCATGTTCA-3′. Amplification was performed in a 20 µl reaction mixture, containing 0.16 mM dNTPs, 0.1 µM forward and reversed primers respectively, 1 mM MgCl_2_, 0.4 U Taq polymerase (Tiangen, China), and 1 µl cDNA. The PCR program consisted of 30 cycles: 94°C for 10 min; 94°C for 1 s; 56–60°C for 30 s; 72°C for 60 s, and the final extension at 72°C for 10 min. Triplicate samples were used for quantitative RT-PCR.

**Table 1:** Detail information about primers used for qRT-PCR variation.

Gene	Forward primer (5′-3′)	Reverse primer (5′-3′)	Tm (°C)	Product (bp)
*KOS*	GTATCTTGGGAGCCGCTG	CATTCTTACGCATGGCAA	56	354
*ABA8OX*	CAAACCCAACACTTTCATC	TTGTCATTTCTACAAAATATTC	54	287
*CLP*	CTCGTACAGAGTCTGGAATT	GTCATTCTTTTTCTGTAGTCC	58	300
*AP2/ERF*	TCGTCTACGTCTTCCTCCAA	TCAATGACATCGTTCCTCG	56	220
*PLP*	ATCCCTGCGGTAGTGCTTC	CATCCTTCTTCGGTGTGGT	55	235
*SnRK1*	GCTCTGTTGGGAAAGGTT	ACTTTTTCATCCATGTCCA	52	356
*GIGANTEA*	CCGAATCCTTTCTCAGCCTAC	CCATAAGCAGCCTCCCAG	53	212
*FD*	AAGTATCATTTTCGACAAACA	GAAGCCGAAGAGTAGTGAG	56	322
*Lox*	GAGTACGAGTACCAGGTGAAG	AGAAGGGATCTTACCAGTCAC	58	263

## Results

### 2.1 Transcriptome Profile during Corm Formation

To investigate the genes involved in corm development, three libraries were constructed from the three stages of corm formation: stolon stage, initial swelling, and swelling stages using the Illumina sequencing platform. Before mapping the tags, the transcriptome of three above stages were preprocessed, and a total of 59909 genes which included 43778 (accounted for 70.07%) CATG site genes used as reference genes were obtained.

Overall, about 6 million tags in each library were obtained with 328156, 273762, and 236840 distinct tags respectively. To get clean tags, all the raw tags were filtered with reference sequences, and 5854021, 5983454, and 5761079 clean tags were obtained including 138319, 116804, and 101739 distinct clean tags in C1, C2, and C3 libraries respectively. Complete data of tags for each library is given in Table 2. The analysis of sequencing saturation was also performed in the three libraries to estimate whether or not the sequenced tags were sufficient to cover the whole transcriptome. We found that the number of detected genes increased until the sequencing tags reached 3 million or more, which indicated that the identified expressed tags were enough to reflect the whole transcriptional information of *Sagittaria trifolia* genome (Fig. S1).

**Table 2:** Categorization and abundance of tags.

		C1	C2	C3
Raw Tag	Total number	6047232	6143854	5899315
	Distinct Tag	328156	273762	236840
Clean Tag	Total number	5854021	5983454	5761079
	Distinct Tag number	138319	116804	101739
All Tag Mapping to Gene	Total number	3096297	2896510	2172619
	Total percentage of clean tag	52.89%	48.41%	37.71%
	Distinct Tag number	65279	54635	46104
	Distinct Tag percentage of clean tag	47.19%	46.77%	45.32%
Unambiguous Tag Mapping to Gene	Total number	3080868	2883680	2165209
	Total percentage of clean tag	52.63%	48.19%	37.58%
	Distinct Tag number	64966	54369	45871
	Distinct Tag percentage of clean tag	46.97%	46.55%	45.09%
All Tag-mapped Genes	Total number	20714	19941	18594
	Percentage of reference genes	34.58%	33.29%	31.04%
Unambiguous Tag-mapped Genes	Total number	20575	19807	18438
	Percentage of reference genes	34.34%	33.06%	30.78%
Unknown Tag	Total number	2757724	3086944	3588460
	Total percentage of clean tag	47.11%	51.59%	62.29%
	Distinct Tag number	73040	62169	55635
	Distinct Tag percentage of clean tag	52.81%	53.23%	54.68%

**Figure 1:**
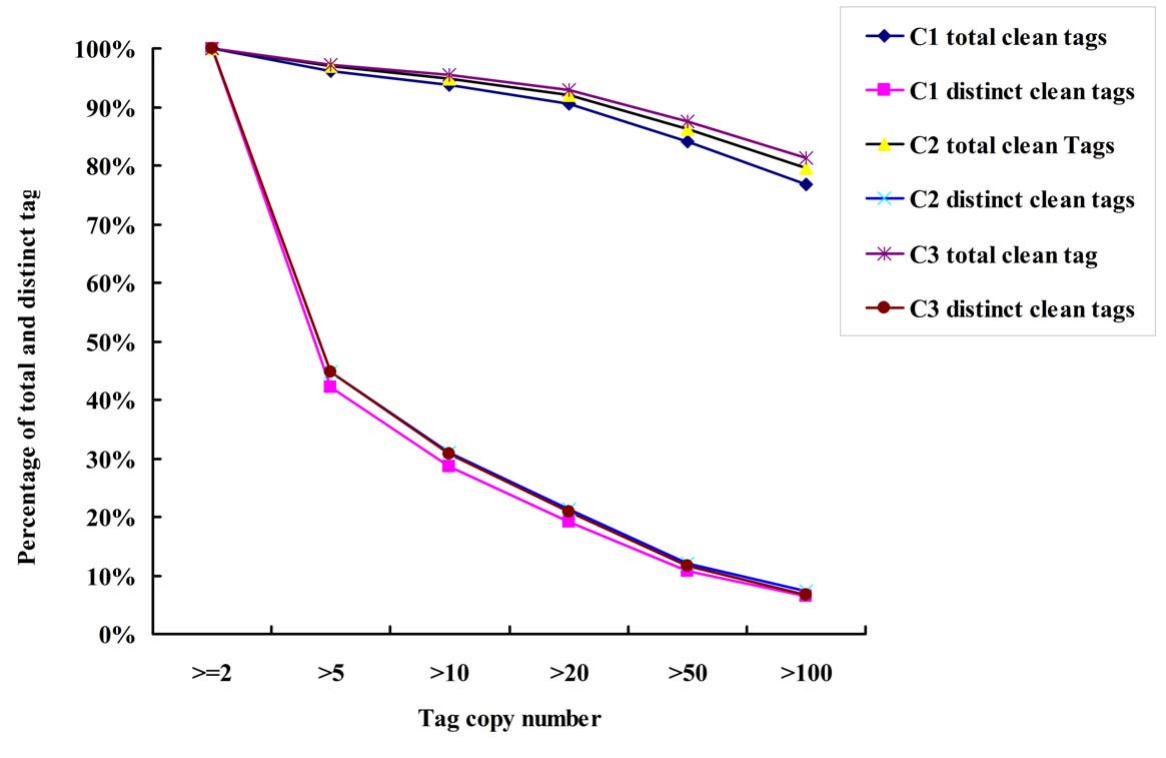
Distribution of total clean tags and distinct clean tags counts from the three libraries.

BLASTx was applied to annotate distinct gene functions by comparison with existing NCBI database using a cut-off E-value of 10^−5^. Among which, approximately 47% of all distinct sequences in three libraries showed an above cut-off BLAST result, and about 53% did hot match with the known genes. These genes could be classified into 26 catalogues according to their functions. One catalogue containing the largest number of genes contained the genes with predicted functions, and the smallest one was related to cell structure (Fig. S2). The copy distribution of total and distinct clean tags in three libraries showed the same tendency with about 5% of distinct clean tags higher than 100 copies and about 30% tags being in 5–50 counts. The number of distinct clean tags between 2–5 copies (approximate 45%) was high as compared with that of others (Fig. 1).

### 2.2 DEGs in Three Libraries

#### Gene expression profiles during corm formation

**Figure 2:**
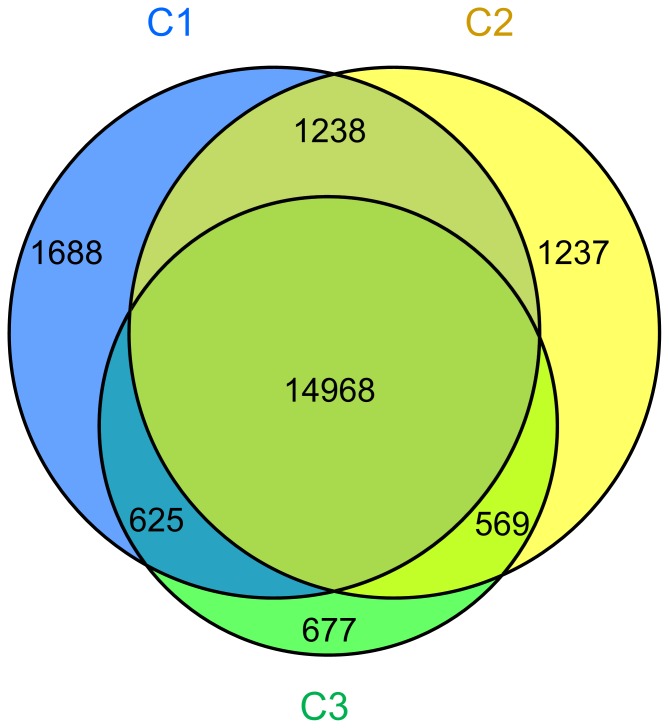
Analysis of tag mapped genes among three libraries.

Three libraries were examined to identify gene expression profiles during corm formation. Total 18519, 18012, and 16839 transcripts were identified from C1, C2, and C3 libraries respectively. Among which, 14968 genes were expressed in all three libraries, 16206 expressed genes were found in both C1 and C2, and 15537 genes existed in both C2 and C3 libraries, respectively (Fig. 2).

DEGs were identified from these three different developmental stages during corm formation to uncover the changes in metabolisms at molecular event. The abundance of transcripts in three libraries was counted by the number of genes per million clean tags (TPM). FDR (false discovery rates) <0.001 and the absolute value of |log2 Ratio|≥1 were used as a threshold to judge whether gene expression was significant. A total of 3011 genes were found to change their transcriptional level in C1/C2 libraries. From which, 1344 were up-regulated (Table S1) and 1667 were down-regulated (data was not shown). In C2/C3 libraries, 2195 genes, including 214 up-regulated genes (Table S1) and 1981 down-regulated genes (data was not shown) altered the expression. A great change was observed in C1/C3 libraries, with changes in transcriptional level of 4643 genes including 616 up-regulated and 3027 down-regulated (Table 3). According to the gene expression profile, a large number of genes showed altered mRNA expression levels in C1/C2 libraries. However, number of transcripts in C2/C3 libraries showed a little change as compared with C1/C2 libraries, suggesting that metabolisms from stolon stage to initial swelling stage were different with that from initial swelling stage to swelling stage.

**Table 3:** DEGs across all libraries.

	C1:C2	C1:C3	C2:C3
Total	3011	4643	2195
Up-regulated	1344	616	214
Down-regulated	1667	3027	1981

All the genes mapped to the reference sequence and genome sequences were examined for their expression differences across the different libraries. Numbers of differentially expressed genes represent transcripts, using threshold values FDR≤0.001 and |log2 Ratio|≥1 for controlling false discovery rates. C1, C2 and C3 represent the samples which were collected at stolon stage, initial swelling stage and swelling stage of corm respectively.

#### Most DEGs in each library

In total, 3011 and 2095 genes altered their expression in C1/C2 and C2/C3 libraries, respectively. Therefore, 20 DEGs with higher levels of changes were selected in C1/C2 and C2/C3 libraries, respectively, to monitor changes in metabolism during corm formation. We found that the most DEGs in C1/C2 libraries belonged to short-chain dehydrogenase/reductase family proteins. DEGs in C1/C2 libraries could be mainly classified into 3 catalogues which belonged to hormone metabolism or response (Ent-kaurene oxidase CYP701A5, Ent-kaurene synthase, CYP707A4 (ABA 8-hydroxylases, AP2/ERF domain-containing transcription factor), energy metabolism (Short-chain dehydrogenase/reductase family protein, Secoisolariciresinol dehydrogenase, and FAD-binding domain containing protein) and stress response proteins (heat shock proteins, wound-responsive family proteins, heavy metal-associated domain containing proteins, anaerobically inducible early genes). In addition, a calmodulin-like protein and myb family transcription factors were also found to be involved in C1/C2 library (Table 4).

**Table 4:** 20 most differentially expressed annotated genes in C1/C2 and C2/C3 libraries based on expressed tag frequency. “−” represents the down-regulation of gene.

Gene ID	Relative abundant (TMP ratio)	Function annotation
**C1/C2** libraries C1/C2 (C2/C3)		
C56569	13.124 (−4.541)	Short-chain dehydrogenase/reductase family protein
C58652	13.056 (−4.493)	FAD-binding domain-containing protein
C59382	13.005 (−6.715)	Ent-kaurene oxidase CYP701A5
C48238	12.972 (−4.783)	Acid phosphatase (APase)
C58140	12.895 (−11.714)	Resistant specific protein-3
C8055	12.215 (−0.127)	ABA 8-hydroxylases
C6524	11.897 (−11.139)	Polygalacturonase-inhibiting protein
C56037	11.725 (−5.967)	3-oxoacyl-[acyl-carrier-protein] reductase
C50776	11.198 (2.514)	Heat shock protein
C18552	10.899 (−10.455)	Ent-kaurene synthase
C54634	10.968 (−3.848)	SMO2-2
C42905	10.568 (−10.171)	Secoisolariciresinol dehydrogenase
C55007	10.498 (1.097)	Temperature-induced lipocalin
C41767	10.478 (−4.034)	Wound-responsive family protein
C57234	10.332 (−4.006)	PRH19
C1731	10.325 (−2.616)	Calmodulin-like protein
C54598	10.112 (1.277)	Calmodulin binding protein
C35117	9.698 (−9.489)	Anaerobically inducible early gene 2
C52384	9.236 (−3)	AP2/ERF domain-containing transcription factor
C17437	9.056 (−1.939)	Myb family transcription factor
**C2/C3** libraries C2/C3 (C1/C2)		
C49391	23.659 (6.998)	Heat shock protein
C8325	20.785 (−92224)	WRKY69
C23809	20.582 (−6.672)	NAD-dependent malate dehydrogenase
C15100	20.258 (7.443)	Oxidoreductase
C55126	19.923 (−5.672)	Pollen Ole e 1 allergen and extensin
C16333	18.255 (−7.098)	Patatin-like protein
C2677	15.241 (0.439)	Nodulin-like protein
C50994	10.362 (−7.267)	K-exchanger-like protein
C55452	7.968 (2.365)	Ribosome biogenesis regulatory protein homolog
C56648	7.835 (−5.672)	Metalloproteinase
C12295	7.725 (−7.267)	Nuclear transport receptor exportin 4
C41931	7.251 (−3.658)	11S globulin precursor isoform 2A
C8797	7.211 (−6.672)	Helicase-like protein
C53039	7.191 (1.365)	Extensin-like protein
C39310	7.135 (−5.672)	Glutathione S-transferase
C37931	7.084 (−5.688)	Calcium:sodium antiporter/cation:cation antiporter
**Gene ID**	**Relative abundant (TMP ratio)**	**Function annotation**
C25514	7.0364 (−9.564)	IAA-amino acid hydrolase ILR1
C5064	7.043 (0.125)	Sucrose non-fermenting related protein kinaseI
C49862	6.709 (4.365)	NAC domain protein
C41150	6.702 (1.937)	GBSSI

In C2/C3 libraries, a great number of genes were involved in storage such as glutathione S-transferase gene, globulin synthesis gene, sucrose synthase, patatin synthesis gene and starch synthesis gene. At the same time, one corm swelling related gene (extension-like protein) and energy metabolism (NAD-dependent malate dehydrogenase) were observed to alter their expression. From above results of gene expression, we conclude that several genes are very important and necessary for corm development in C1/C2 stages, such as ERF domain-containing transcription factor, genes relevant to hormone metabolism (Ent-kaurene oxidase CYP701A5, Ent-kaurene synthase and ABA 8-hydroxylase), calmodulin-like protein and calmodulin binding protein (Table 4). It was observed that a lot of genes were down regulated in C1/C2 and C2/C3 libraries, most of which in C1/C2 libraries were involved into synthesis of substances, and others in C2/C3 libraries were relevant to cell growth and differentiation, translation, RNA process and modification (data not shown).

Some transcription factors were also identified which are important in regulating the gene expression during corm formation. We summarized all the up-regulated transcriptional factors in C1/C2 and C2/C3 libraries. A total of 19 and 14 transcription factors were found in C1/C2 and C2/C3 libraries, respectively (Table 5). In C1/C2 libraries, some of transcription factors were ethylene responsive proteins (AP2/ERF, ERF2b and ERF2), Myb, CaM-binding transcription factor, WRKY45, NAC, bZIP, NPR1-like protein, and bHLH transcription factors. In C2/C3 libraries, expression levels of many important regulators (e.g. ERF3, HSP8, MYB, CKB3, SCL11, JMJC, bHLHb, ATAIB, GPRI1, and others) were enhanced, most of which are involved in storage metabolism and stress response (Table S1).

**Table 5:** Expression abundance of up-regulated transcriptional factors during corm formation.

Gene ID	Relative abundant (TMP ratio)	Function annotation
**C1/C2**		
C52384	9.108	AP2/ERF domain-containing transcription factor
C17437	8.870	Myb family transcription factor
C24919	8.840	Transcription factor RF2b
C3379	6.643	CaM-binding transcription factor
C57104	6.510	bZIP transcription factor bZIP17
C12669	5.643	WRKY45 transcription factor
C47594	5.425	Transcription factor jumonji domain-containing protein
C13894	5.044	Transcription factor PCF8
C8257	5.125	Transcription factor
C17055	5.118	Transcription factor HY5
C12723	5.101	bHLH transcription factor PTF1-like
C43302	5.098	MADS box transcription factor
C53480	5.044	Ethylene responsive transcription factor 2b
C18355	3.051	ERF2 transcription factor
C58603	2.981	Arabidopsis NAC domain containing protein
C18200	1.912	Regulatory protein NPR1-like
C36044	1.493	Scarecrow-like transcription factor 11
C9570	1.071	DNA binding/protein binding/transcription factor
C47273	1.007	LIM transcription factor homolog
**C2/C3**		
C59162	8.022	Heat shock factor protein HSF8
C12731	6.108	DNA binding/transcription factor
C54729	5.725	Scarecrow-like transcription factor 11
C49297	5.700	Myb family transcription factor
C58385	5.899	CKB3; protein kinase regulator
C18736	5.689	Transcription factor jumonji domain-containing protein
C1441	5.589	Similar tp transcription factors
C52124	5.425	Transcription factor AP2-EREBP
C32284	5.256	GBF’s pro-rich region-interacting factor 1
C9294	2.074	PHD finger transcription factor-like
C44773	1.138	Ethylene-responsive transcription factor
C12005	1.072	Basic helix-loop-helixfamily protein
C51733	1.064	ABA-inducible BHLH-type transcription factor
C18668	1.012	DRE transcription factor 1

#### Genes related to corm formation

The data sets of this experiment were compared to those reported previously with the aims to test whether the transcription patterns had coverage of the well-defined genes. We found that 16 identified genes relevant to corm formations were found to change their transcriptional level. The expression of these genes and their biological functions are listed in Table 6. Among which, 8 genes including flowering locus D, zinc finger CONSTANS-like protein, SFT1 family, cycling dof factor, BEL1-like HD transcription factor, sucrose synthase, Ca^2+^/Calmodulin-like protein, and lipoxygenase enhanced their transcriptional levels, and 1 gene photoperiod sensitivity 5 (SE5) did not show any significant change in its expression in all the three libraries. Seven genes (GIGANTEA, MADS-box transcription factor, FRUITFUL-like protein, Class I knox protein, Knox protein partner, ADP/ATP translocator-like, AFB auxin receptor protein PintaTIR1) directly decreased their expression during the corm formation.

**Table 6:** Expressed abundance of some corm formation related genes identified previously.

Gene ID	Assetion	TPM-C1	TPM-C2	TPM-C3	Function annotation	References
C4528	gi|240255318	0.01	2.17	4.52	FD (Flowering locus D)	Sarkar 2008
C9851	gi|115440895	0.85	73.2	76.4	zinc finger CONSTANS-like protein	Martinez-Garcia et al. 2002
C5936	gi|145712966	19.3	5.52	5.03	GIGANTEA (clock-regulated protein)	Abelenda et al. 2011
C792	gi|145617255|	0.34	0.01	0.01	MADS-box transcription factor	Hannapel et al. 2004
C12711	gi|157674589	0.51	0.34	0.01	FRUITFUL-like protein	Abelenda et al. 2011
C54289	gi|159479168	12.13	20.56	16.49	SFT family	Krieger et al. 2010
C59218	gi|15231491	24.77	24.44	20.14	photoperiod sensitivity 5 (SE5)	Tsuji et al. 2010
C9570	gi|15232818	4.61	9.69	1.39	Cycling Dof Factor	Imaizumi et al. 2005
C48167	gi|84453182	1.37	5.34	1.22	BEL1-like HD transcription factor	Banerjee et al. 2006
C5539	gi|258958638	44.41	23.9	10.07	knox protein	Hay and Tsiantis 2010
C58867	gi|1814234	45.78	25.08	36.45	Knox protein partner	Chen et al. 2004
C22645	gi|78191448	182.99	198.21	80.71	ADP/ATP translocator-like	Tjaden et al.1998
C59398	gi|55741123	0.51	3.34	1.01	Sucrose synthase	Fernie and Willmitzer 2001
C17314	gi|115459158	0.01	4.35	8.33	Ca^2+^/Calmodulin-like protein	Kim et al. 2009
C51000	gi|32454708	0.01	0.33	0.02	Lipoxygenase	Koloniets et al. 2001
C1771	gi|258676531	12.98	9.53	4.69	AFB auxin receptor protein pintaTIR1	Nishimura et al. 2009

TMP, transcripts per million clean tags.

### 2.3 Expression Analysis of Nine Genes through qRT-PCR

**Figure 3:**
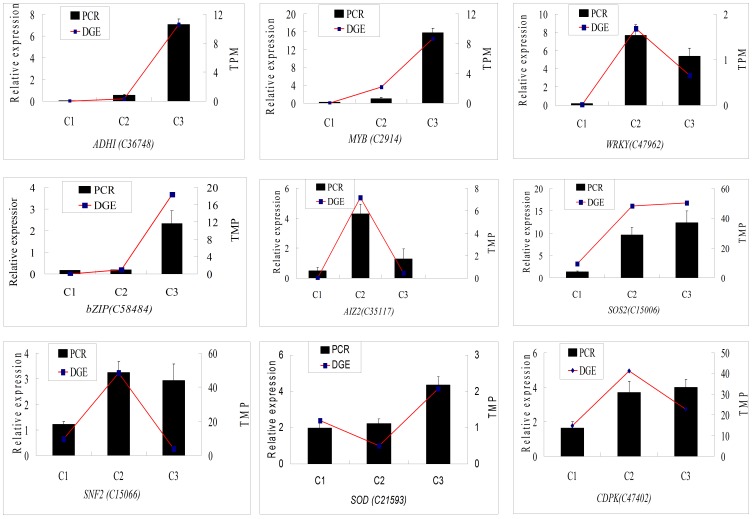
Validation of tag-mapped genes from three stages of *Sagittaria trifolia* with quantitative RT–PCR.

To investigate the changes of gene expression at the mRNA level, we performed quantitative RT-PCR for nine genes which include: Ent-kaurene oxidase (KOS), ABA 8-hydroxylases (ABA8OX), Calmodulin-like protein (CLP), AP2/ERF domain-containing transcription factor (AP2/ERF), Patatin-like protein (PLP), Sucrose non-fermenting related protein kinaseI (SnRK1), FD, zinc finger CONSTANS-like protein and Lipoxygenase (LOX), which are involved in corm formation. Overall, expression analysis of eight genes for the three developmental stages i.e. stolon, initial swelling, and swelling stage by qRT-PCR showed similar tendency as found in Tag-sequencing, which indicates a correspondence of the results from qRT-PCR analysis with the Tag sequencing analysis. Only one gene (*FD*) was observed with some difference in transcriptional level between Tag-sequencing analysis and qRT-PCR data (Fig. 3). The expression of *FD* with qRT-PCR analysis showed no significant change in C1/C2 libraries, whereas it showed some enhancement in C2/C3 libraries.

## Discussion

### Tag-sequencing Based Analysis of Transcriptome Expressed during Corm Formation

High throughout tag-sequencing is being applied to study plant development, and many regulatory mechanisms at molecular level have been identified [34], [35], [37], [38]. Tag-mapped genes have been testified to fully cover the whole plant genome, although a large number of genes have not been annotated [35]. In this study, genes which showed differential expression during corm formation were analyzed using tag-sequencing technique. Approximately 6.0 million tags were identified per library, and 5854021, 5983454, and 5761079 clean tags were obtained. Only 20575, 19807, and 18438 genes from C1, C2, and C3 libraries, respectively, could be annotated, due to unavailability of complete genome of *Sagittaria trifolia* (Table 2). We found that many genes changed their transcriptional level significantly, during corm formation. The number of DEGs in C1/C2 libraries was more than that of C2/C3 libraries (Table 3), suggesting that regulations from stolon stage to initial swelling stage were more complex as compared with that of initial swelling stage to swelling stage.

### Identification of DEGs in C1, C2, and C3 Libraries

DEGs in three libraries were involved in the transport signal transduction, small molecular biosynthesis, transcription, cell cycle, response to the stimuli, organelle organization, anatomical structure development, cell differentiation, translation, organ development, cellular macromolecular metabolic processes, energy, and cellular component organization (data not shown). Among 20 DEGs in C1/C2 libraries with higher levels of changes, are involved in hormone synthesis and response, energy metabolism, and response to stresses. In C2/C3 libraries, however, the expression profiles of major DEGs were relevant to the formation of storage organ. All genes enhanced expression in C1/C2 and C2/C3 were listed in Table S1.

### Energy Metabolism

Most species of *Sagittaria trifolia* are damaged when submerged under water for long periods [39], therefore, *Sagittaria* must adapt to oxygen shortage during growth. This might be the major reason why many submergence responsive genes were found to improve the expression in C1/C2 libraries, such as *adh* (C36748), *myb* (C17437), *WRKY* (C12669), *bZIP* (C57104) and anaerobically inducible early gene (C35117) (Table 4, Table S1). It was reported that energy regulation was a measure to alleviate the hurt acquired because of submergence [40], [41], [42], and at the same time, many critical processes for storage organ formation also need energy consumption [43]. Thus special regulation of gene expression can provide enough energy and metabolic components to support the need of developing storage organ.

ATP is normally generated from glycolysis in plant cell to sustain plant growth. For generation of ATP, ATP/ADP translocator (C48129) is thought to be a gated pore through which ADP and ATP are exchanged between the mitochondrial matrix and the cytoplasm. ATP/ADP translocator is an inner membrane protein which is observed to enhance the tuber number and changes tuber morphology in transgenic plant [44]. Evidence shows that delivery of NADPH, NADH, reduced ferredoxin and ATP, in the specific cellular compartments will happen for energy-consuming reactions due to limitation of pool sizes of these energy carriers. Generally, NADPH, NADH and reduced ferredoxin are not directly transported across bio-membranes, therefore, indirect transport of reducing equivalents is achieved by malate/oxaloacetate shuttles, involving malate dehydrogenase (MDH) and NADP-MDH (Isoenzyme of MDH) for the interconversion of oxaloacetic acid and malate [45], [46]. MDH is one of the more active enzymes in peroxisomes, mitochondria, chloroplast, glyoxysomes, and cytoplasm. The activities of the enzymes of malate and NADP-MDH valves was changed when plants are subjected to conditions such as high light, high CO2, nutrition or stresses.

A lot of evidences show that NADP-MDH is involved in response to environmental factors. When plants are subjected to stress conditions, which require changed activities of the enzymes of malate valves, changed expression levels of MDH isoforms can be observed. Hameister et al. (2007) analyzed transcriptional regulation of NADP-MDH in *Himatanthus sucuuba* and in some plants from Brassicaceae, the amount of transcripts increases twofold after transferring to stress condition [47]. Monitoring the expression changes in NADP-MDH after a transfer into conditions of persisting over-reduction, i.e. a transfer of Arabidopsis plants into high light levels and moderately decreased temperature, Becker et al. (2006) find found that both transcript and protein amount of NADP-MDH are up-regulated within a few hours [48]. NADP-MDH activity is nearly tenfold higher on a chlorophyll basis in cold-grown winter wheat, as compared to control plants grown with low temperature stress. When facultative CAM plants, *Mesembryanthemum crystallinum* and rice are exposed to salt stress, the total NADP-MDH activity is also higher as compared with the control conditions [49], [50]. It is evident from the above studies that an increase in NADP-MDH activity during the corm formation probably had two important roles: one is provision with the energy for corm development and the other is to promote adaptation of plant to submergence. In addition, FAD binding containing protein (C58652), ATPase (C24284), phosphoenolpyruvate carboxylase (C59661) and pyruvate dehydrogenase (C54727) were also observed to improve expression during this process. In this study, the expression of ATP/ADP translocator had no novel change in C1/C2 libraries, and was decreased in C2/C3 libraries, therefore, enhanced expression of MDH (C28383) and NADP-MDH (C58557) might be an adaptive response for *Sagittaria trifolia* to against submergence.

### Carbohydrate/storage Metabolism

Starch is the most important component found in the corm (storage organ) of *Sagittaria trifolia*. It is reported that swelling of storage organ and accumulation of starch occur simultaneously [51]. Therefore, synthesis of starch shows high coordination with formation of storage organ [52]. In this experiment, the expression of a gene encoding granule-bound starch synthase was enhanced during the corm development (Table 4). Granule-bound starch synthase is believed to have a higher amounts of starch synthesized in plant storage organs [53].

Until now, two kinds of granule-bound starch synthase (*GBSSI* and *GBSSII*) exist in the plant kingdom with different expression profiles. In rice, the content of amylose is increased in transgenic plants containing *GBSSI* as compared with that of the control plants. Further examination shows that the difference in amount of amylose in transgenic and non-transgenic plants results from long unit chains of amylopectin, which is similar to the report of Hanashiro et al. (2008) [54]. The DEG found in our study belonged to *GBSSI* (C41150), whereas we did not find *GBSSII* gene expression due to two possible reasons: either *GBSSII* does not exist in the *Sagittaria* genome; or *GBSSII* was not expressed in the corm during the developmental period.

Patatin, which is identical to glycoprotein, is usually believed as a major storage protein in corm, tuber, rhizome, and bulb. We found that expression of *patatin* (C16333) decreased in C1/C2 libraries, and enhanced in C2/C3 libraries. The above result implied that patatin accumulated in the swelling stage. In some species of potato, 40% soluble tuber protein is derived from patatin. Compared with other storage proteins, patatin is more stable because no degradation products are detected during tuber development. Evidence shows that *patatin* might be involved in metabolism because acyl hydrolase has been found to be encoded by this gene [55], [56]. Another study shows that *patatin* is involved in pollen development [57], [58], which is further testified by Vancanneyt et al. (1989). [59]. Perl et al. and Bamfalvi et al. (1994) have documented the function of patatin which is associated with early events of formation of storage organ underground according to its expression profile [60], [61]. A small amount of patatin is found in stolon tips from non-induced plants, but rapidly accumulates during tuberization [62]. In addition, patatin is observed to be synthesized only in stolon and tuber, and its accumulation has high correlation with tuber swell [63]. Rocha-Sosa et al (1989) observed that the expression of *patatin* in non-tuber tissues is induced by sucrose, although sucrose is not believed to directly regulate patatin gene expression [64], [65].

Accumulation of sugar through photosynthesis is the most fundamental event in the whole life of plant, because it supports plants to adjust some physiological activities and provide enough materials and energy to complete those activities [66]. Therefore, the processes of sugar synthesis, transport, consumption, and storage have been widely studied in past years [67]. It has been testified that soluble carbohydrates, most notably sucrose, have convincingly been described to be strong inducers for formation of underground storage organ [68], because increasing concentration of sucrose in medium during cultivation leads to more numbers of tubers [12]. Sucrose leading to more tuber numbers mainly presents its role as an inducing signal molecule and increase in the level of sucrose in stolons results in an increased number of initiated tubers [69]. We found that expression of gene (Sucrose synthase: C59398) involved in sucrose synthesis was enhanced at transcriptional level in C1/C2 libraries (Table S1), suggesting that accumulation of sucrose is helpful for corm formation of *Sagittaria*. Further evidence also shows that SNF1 kinase (the sucrose non-fermenting-1) is involved in sugar-signaling pathways to regulate metabolism of carbohydrate or other storage proteins [70]. From the characteristics of gene expression, SNF1 (C50647) showed enhanced expression, which undoubtedly promoted the formation and development of corm (Table 4, Table S1).

### Up-regulation of Transcription Factors during Corm Formation

Gene expression is regulated by transcription factors which is a crucial part of the plant response to control the entire metabolic processes. Expression levels of 19 and 14 transcription factors were enhanced in C1/C2 and C2/C3 libraries, respectively (Table 5). For these transcription factors, we found that CaM-binding protein and AP2-EREBP (C52124) have been identified to play critical roles in the formation of underground storage organ. Ca^2+^ has been developed as a second messenger to perceive endogenous and exogenous signals before system responses [71]. Calmodulin is characterized as Ca^2+^ sensor, and a variety of cellular processes are modulated after Ca^2+^ binds to CaM [72]. Ca^2+^/CaM have already been testified to be involved in the formation of storage organs [73]. Further study shows that overexpression of a CaM gene (*PCM1*) in potato plants produces more elongated tuber [74]. In addition, CaM-binding proteins have been found in many plants and their functions have also been identified that they are involved in the development of underground storage organs [71], [75].

A potato CaM-binding protein (PCBP) is found to play an important role in signaling transduction during tuber formation according to characteristics of its expression [76]. We observed that calmodulin-like protein (C17314) and calmodulin binding protein (C54598) have similar expression profiles in C1/C2 libraries and C2/C3 libraries. From stolon to initial swelling stage of corm, expression levels of these two genes improved, and decreased from initial swelling stage to swelling stage, indicating that Ca^2+^/CaM might be involved in processes from stolon stage to initial swelling stage of corm.

In this study, the expression level of ethylene responsive factor was found to be enhanced (Table 5). From the characteristics of expression, we could conclude that this gene play an important role in corm formation. Ethylene is not only involved in a range of biotic and abiotic stress responses, especially to help wetland plants to adapt to anaerobic condition, but also mediates swelling of underground storage organs [77]. At the same time, AP2/EREBP gene (Ethylene-responsive element binding proteins), which is unique in plant kingdom also shows multi-functions in metabolism from response to stresses to regulation of plant development [78]. Overexpression of *StEREBP* confers potato plant more resistance to abiotic stress, whereas down-regulation of *AP2/EREBP* leads to a series of other effects such as decreased cell size, plant height, hypocotyl elongation and fertility [79].

In addition, several AP2 members are found to be involved in controlling flowering time [80]. Cernac and Benning (2004) reports that a putative AP2/EREBP transcription factor, *WRI1* controls the seed storage metabolism in Arabidopsis, because overexpression of this gene causes enhancement in the oil content of seed and triacylglycerols in developing seedlings [81]. Overexpression of an *EREBP* in rice can increase the expression of waxy gene which directly results in a change of storage content [82]. Evidence shows that AP2/EREBP is involved in abscisic acid and sugar signal transduction pathway according to its expression [83]. Therefore, future studies should also be focused to identify the role of AP2/EREBP in corm formation with transgenic techniques.

### Hormonal Regulation

Swelling of underground storage organ is affected by various environmental and endogenous factors. Short photoperiods, low temperatures, low nitrogen and hormone favor the formation of storage organ [68]. In literature, many reports describe the importance of gibberellic acid (GA), abscisic acid (ABA), IAA, ethylene for the formation of storage organ. GA content is enhanced in transgenic plants by overexpression of GA oxidase gene, and elevation of GA content leads to transgenic potato plants that require a longer duration of short-day photoperiods to form tubers. Whereas, inhibition of this enzyme activity results in earlier tuberizing as compared with that of control plants [24].

A dwarf mutant of *S. tuberosum* ssp. *Andigena* showed a decrease in GA content which could tuberize both in LD and SD condition. However, when GA biosynthesis is inhibited, plants cannot tuberize in SD [18]. These results show that GA is probably involved in the photoperiodic induction to regulate the formation of storage organ. In addition, Xu et al. (1998) [84] observed that high content of GA promotes stolon elongation and inhibit corm formation. In this study, ent-kaurene oxidase CYP701A5 and ent-kaurene synthase (18552) involved in GA biosynthesis pathway enhanced the transcriptional level in C1/C2 libraries, whereas it decreased in C2/C3 libraries (Table 4, Table S1). Enhancing GA level in C1/C2 libraries probably benefited the elongation of stolon and decreased GA level in C2/C3 libraries and might have benefited the corm swell.

Several studies have been performed to study the effects of ABA on the swell of underground storage organ. Application of exogenous ABA increases endogenous ABA level of plant under tuber-inducing conditions, which results in the earlier initiation of tuber, the formation of tubers and enhancement of tuber number [85], [86]. Same phenomenon is also found when plant is promoted to form tuber when exogenous ABA is applied [87]. Xu et al. (1998) [84] observed that exogenous ABA stimulates tuberization and reduced stolon length under normal growth condition. However, Hussey and Stacey [88](1980) found that tuberization is blocked by ABA, and exogenous ABA prolongs the process of tuber formation. The expression of a gene encoding ABA hydroxylase (C8055) was enhanced in C1/C2 libraries and the enhanced transcriptional level undoubtedly decreased content of ABA in initial swelling period, which indicates that low ABA content benefited corm formation of *Sagittaria trifolia*. In addition, the expression of some identified corm-related genes was also documented in this study (Table 6), and most of these genes were relevant to flowering and hormone response. Overall, according to the gene expression profiles, we conclude that formation of corm was regulated by multiple genes.

### Conclusions

Gene expression of three libraries with different developmental stages for corm formation was studied using high-throughput tag-sequencing based on Solexa Genome Analyzer Platform. Based on results of tag-sequencing, 20575, 19807, and 18438 tags were annotated in three stages (stolon stage, initial swelling stages and swelling stage) respectively after comparison through the existing databases. In addition, a number of important differentially expressed genes relevant to corm development were found from these three libraries. Quantitative RT-PCR for nine genes was used to identify the results of tag-sequencing, and the results revealed that gene expression by qRT-PCR showed high correlation to the Tag sequencing analysis.

## Supporting Information

Figure S1 **GO analysis of genes expressed during the corm formation.** All the genes identified in C1/C2 and C2/C3 libraries were classified into 26 classifications according to gene functions
(TIF)

Figure S2 **Sequencing saturation analysis of three libraries.** C1: tag-sequencing for stolon stage; C2: tag-sequencing for initial swelling stage; C3: tag-sequencing for swelling stage
(TIF)

Table S1 **Genes were found to enhance their expression in C1/C2 and C2/C3 libraries.** These genes were listed in descending order according to alteration of expression during corm formation.
(XLS)
