# Supplementary figures and images for: Identification of Differentially Expressed Genes Relevant to Corm Formation in Sagittaria trifolia
Source: PLoS One. 2013 Jan 24;8(1):e54573. doi: 10.1371/journal.pone.0054573 (PMC3554737; doi:10.1371/journal.pone.0054573)

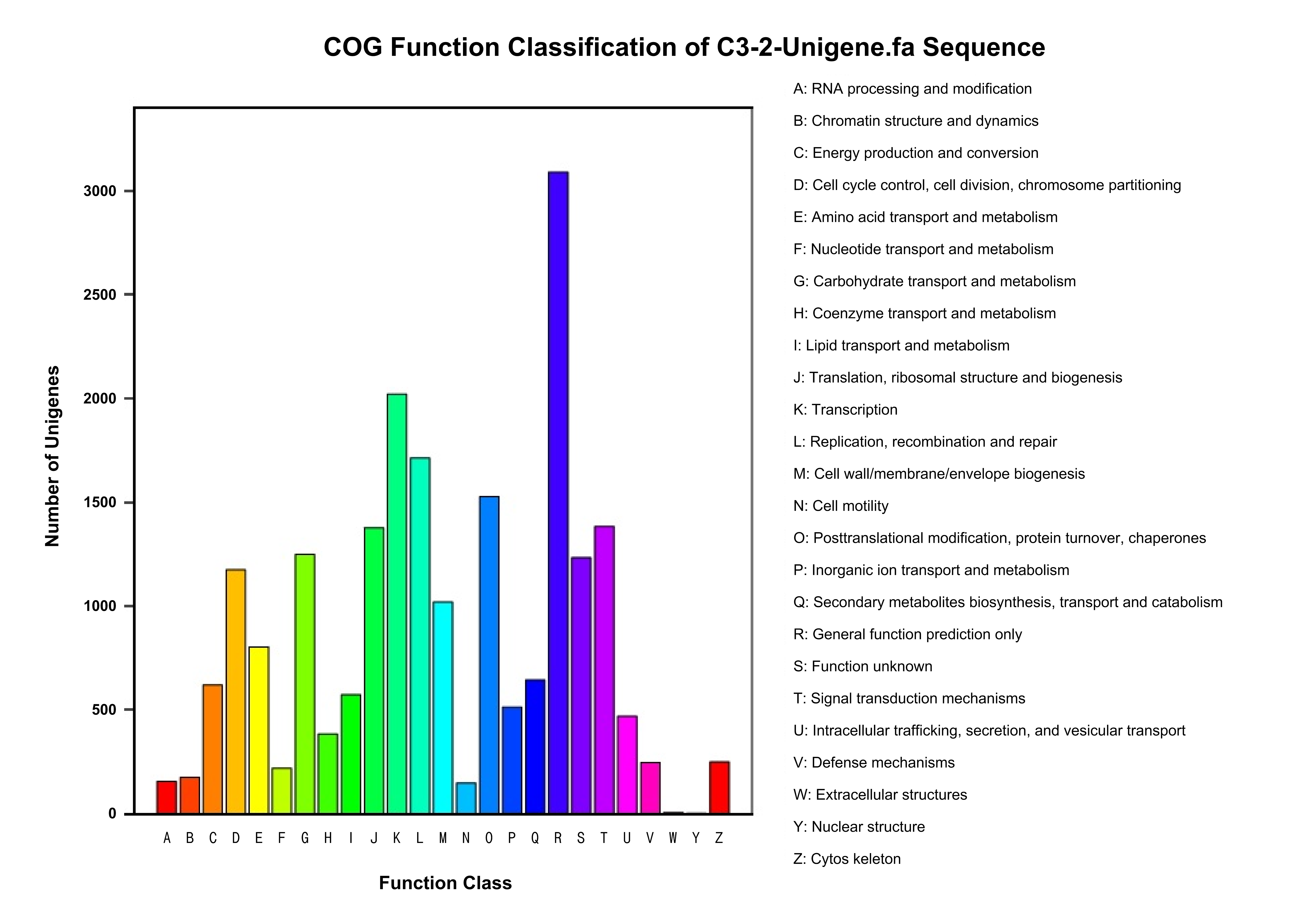

Supplement: Figure S1 — GO analysis of genes expressed during the corm formation. All the genes identified in C1/C2 and C2/C3 libraries were classified into 26 classifications according to gene functions (TIF) [file pone.0054573.s001.tif]

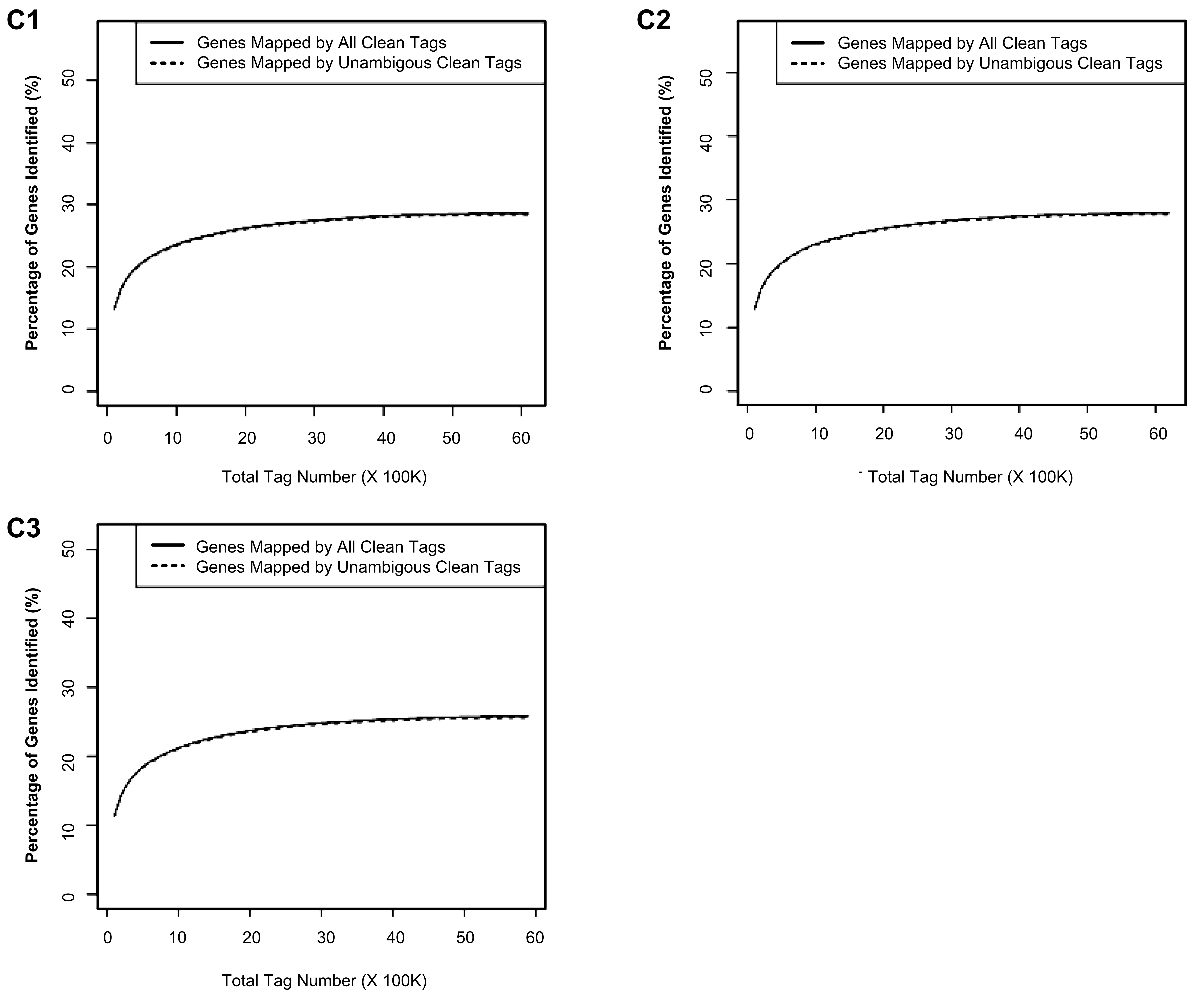

Supplement: Figure S2 — Sequencing saturation analysis of three libraries. C1: tag-sequencing for stolon stage; C2: tag-sequencing for initial swelling stage; C3: tag-sequencing for swelling stage (TIF) [file pone.0054573.s002.tif]
